# Improvement of Source Number Estimation Method for Single Channel Signal

**DOI:** 10.1371/journal.pone.0164654

**Published:** 2016-10-13

**Authors:** Zhi Dong, Junpeng Hu, Bolun Du, Yunze He

**Affiliations:** 1 College of Mechatronics and Automation, National University of Defense Technology, Changsha 410073, Hunan Province, China; 2 College of Electrical and Information Engineering, Hunan University, Changsha, 410082, China; West Virginia University, UNITED STATES

## Abstract

Source number estimation methods for single channel signal have been investigated and the improvements for each method are suggested in this work. Firstly, the single channel data is converted to multi-channel form by delay process. Then, algorithms used in the array signal processing, such as Gerschgorin’s disk estimation (GDE) and minimum description length (MDL), are introduced to estimate the source number of the received signal. The previous results have shown that the MDL based on information theoretic criteria (ITC) obtains a superior performance than GDE at low SNR. However it has no ability to handle the signals containing colored noise. On the contrary, the GDE method can eliminate the influence of colored noise. Nevertheless, its performance at low SNR is not satisfactory. In order to solve these problems and contradictions, the work makes remarkable improvements on these two methods on account of the above consideration. A diagonal loading technique is employed to ameliorate the MDL method and a jackknife technique is referenced to optimize the data covariance matrix in order to improve the performance of the GDE method. The results of simulation have illustrated that the performance of original methods have been promoted largely.

## Introduction

The problem of single channel source number estimation (SCSNE) is being widely investigated in many fields, e.g., image processing, fiber communication [1–3], nondestructive testing [4] and single channel blind signal separation (SCBSS). In recent years, SCBSS has been considered as one of the most challenging research topics in these areas [5–8]. Numerous algorithms have been carried out to solve the SCBSS problem, such as multiple signal classification (MUSIC)[9], estimating signal parameters via rotational invariance techniques (ESPRIT)[10], and independent component analysis (ICA)[11]. However, the performance of these blind source separation (BSS) algorithms and signal spectrum estimation methods could be significantly deteriorated with inaccurate estimation of source number.

In order to process the single channel signals, it is acceptable to expand the data dimensions and convert the single channel data to a multi-channel form. Then many long-tested array signal processing algorithms can be used for references. Nowadays, multiple sampling method[12], the signal sparse representation[13], delay process [14] and many other methods can be used to extend the size of the data. Among them, the multiple sampling method needs cooperation of the data sample front end. Sparse decomposition method costs a high computational complexity, and not all signals are provided with sparse characteristics. Delay process is relatively simple and low complexity; however it needs relatively more snapshots.

Many excellent algorithms of source number estimation have been found in the literatures, such as the methods based on hypothesis testing method [15, 16], information theoretic criteria (ITC) based approaches [17] and Gerschgorin’s disk estimation (GDE) method [18]. The hypothesis testing based method has to set an artificial threshold, which does not constitute an easy decision under some circumstances. Methods based on ITC are established upon the differences between eigenvalues of signal and noise, mainly represented by Akaike information criteria (AIC) [17] and minimum description length (MDL) [19]. Although the computational complexity is relatively low, they can not work with colored noise. GDE method can deal with the colored noise, but its performance exacerbates at low SNR condition.

In addition to the above methods, Keyong Han proposed a method which is based on jackknife technique for array signal source number estimation [20]. The jackknife technique was introduced to reduce the estimator bias in the general context by Quenouile [21]. It is a resampling method that is most frequently used [22]. It has received widely researched in many applications because of its simplicity [22–25]. A method has been proposed which exploits eigenvectors instead of sample eigenvalues to detect the source number in array signal processing [26]. Nonetheless, it is not quite appropriate for the single channel application. Jayme G. A. Barbedo presented a two-stage procedure to determine the number of sources present in a single-channel music signal [27]. Lei Huang proposed a MMSE-Based MDL source number estimator with the prior knowledge of training sequence [28].

Although the AIC method achieves high detection performance at low SNR, it is not consistent. Owing to its properties of simplicity and consistency, the MDL estimator has become the standard tool for estimating the number of sources [29]. This paper detailed the application of MDL and GDE methods to estimate the source number of single channel received signal. The single channel data is converted into a multi-channel form by delay process. Simulation results indicate that the MDL method cannot estimate the source number effectively when the received signal is contained with colored noise. The GDE method underperformance at low SNR, though it can manage with colored noise. To improve the detection performance of these methods, a diagonal loading technique is introduced for the MDL method and the jackknife technique is used to optimize the data covariance matrix for the GDE. The simulation results show that the method put forward in this paper can effectively get the source number of the single-channel received signal and the betterment presented in this paper obtains a remarkable progress.

The remainder of this paper is structured as follows: Section 2 introduces the model of the single channel received signal. Section 3 shows the single channel dimension expansion method. Section 4 describes the source number estimation algorithms and the improvement approaches proposed by this paper. Section 5 shows the experimental results to verify the performance of the improved methods, which is followed with the conclusions in Section 6.

## Signal Model

A multi-channel received signal model with *p* channels and *q* source signals is outlined as follow [30–33].

x(t)=As(t)+n(t) (1)

**x**(*t*) = [*x*_1_(*t*), …, *x*_*p*_ (*t*)]^*T*^ is the observed data. **A** = [*a*(*θ*_1_), …, *a*(*θ*_*q*_)], **s**(*t*) = [*s*_1_(*t*), …, s_*q*_ (*t*)]^*T*^ and **n**(*t*) = [*n*_1_(*t*), …, *n*_*p*_ (*t*)]^*T*^ are the steering matrix, *q* × 1 source waveform vector and noise vector, respectively. The noise covariance is expressed as $\sigma_{n}^{2}I$. $\sigma_{n}^{2}$ is an unknown scalar and **I** is an identity matrix.

While in the single channel condition, only one data can be observed at every time point. The signal model becomes as[34, 35]

X(t)=AS(t)+N(t) (2)

where *X*(*t*) = [*x*(1), *x*(2), …, *x*(*m*)] represents the observed data, *t* indicating the observation time. **A** = [*a*_1_, *a*_2_, …, *a*_*m*_] is the mixing matrix. *S*(*t*) = [*s*_1_(*t*), *s*_2_(*t*), …, *s*_*m*_(*t*)]^*T*^ is a source signal matrix. Noise and signal are independent. The parameter estimation of single channel signal is an underdetermined problem. It is difficult to process the single channel signal directly.

On the other hand, the estimation of source number in array signal processing is relatively simple and convenient. Therefore, it is a considerable approach to convert the single channel signal to an array signal processing problem.

## Construction of Multi-Channel

The single channel received data is a one dimensional matrix. In order to make use of the multi-channel source number estimation algorithms, it is essential to expand the dimension of the data matrix. This paper uses delay process to construct the multi-channel data matrix from the single channel received data. Assuming that the single-channel data are denoted as *y*(*n*), *n* = 1, 2, …, *L*, the received signal can be expressed as follow with delay process.

$$y_{i}=y(n+(i-1)d)$$ (3)

Therefore, a *N* channels received data is formed.

$$Y=\left[\begin{array}{c} y(n)\\ y(n+d)\\ \vdots \\ y(n+(N-1)d) \end{array}\right]$$ (4)

where *d* describes the delay length of each channel. *N* is the total number of channels, which could not be less than the number of sources.

The data of each constructed channel should be passed through a filter which intends to have a more realistic respond of received channel. Set the frequency response of each filter as

$$H_{i}(e^{j\omega })=\left|H_{0}(e^{j\omega_{0}})\right|\cdot e^{j\varphi_{i}(\omega )}i=1,2,\ldots ,N$$ (5)

where, |*H*_0_ (*e*^*jω*_0_^)| represents the amplitude frequency response of a prototype FIR filter. And *φ*_*i*_(*ω*) denotes phase frequency response of each channel. Therefore, the final multi-channel data matrix can be expressed as follow.

$$Y=\left[\begin{array}{c} y(n)*h_{1}\\ y(n+d)*h_{2}\\ \vdots \\ y(n+(N-1)d)*h_{N} \end{array}\right]$$ (6)

where, *h*_*i*_, *i* = 1, 2, …, *l* are the impulse response functions of the filters for each channel. With the process mentioned above, a single channel received signal with 1 × *L* received data is transformed to a multi-channel signal with *N* × (*d* + *l*– 1) dimensions.

## Source Number Estimation Algorithms

### MDL method

Assume that the noise and source signals are mutually independent and unrelated. Then the covariance matrix of the observed signal can be rewritten as follow.

$$R_{X}=E\left\{X(t)X^{T}(t)\right\}=AR_{S}A^{T}+\sigma^{2}I$$ (7)

where, *R*_*s*_ = *E*{*S*(*t*)*S*^*T*^ (*t*)} is the signal covariance matrix and σ^2^*I* represents the covariance matrix of the noise. Transform *R*_*X*_ by eigenvalue decomposition (EVD) to obtain its eigenvalues.

$$R_{X}=\sum_{i=1}^{p}\lambda_{i}u_{i}u_{i}^{H}$$ (8)

where, *λ*_*i*_, *i* = 1, …, *p* are the eigenvalues and *u*_*i*_, *i* = 1, …, *p* are the corresponding eigenvectors.

On account of the noise and signal are independent, the eigenvalues of *R*_*X*_ can be decomposed as *λ*_1 =_ *μ*_1_ + *σ*^2^, *λ*_2 =_ *μ*_2_ + *σ*^2^, …, *λ*_*n*_ = *μ*_*n*_ + *σ*^2^, *λ*_*n*+1_ = *λ*_*n*+2_ = ⋯ = *λ*_*m*_ = *σ*^2^. Ideally, the eigenvalues of signal are far greater than that of noise. The source number is equal to the amount of the eigenvalues which are larger than a preset threshold.

However, due to the limited number of snapshots and the impact of multipath propagation, the eigenvalues of noise and signal are promiscuous. Some criteria have to be introduced to estimate the number of sources precisely. MDL is utilized to select the model depending on the concept of the shortest code length, proposed by Rissanen [19, 36]. The estimation function of MDL can be show as follow.

$$MDL=-\text{log}f(Χ|\hat{\theta })+\frac{1}{2}k\text{log}N$$ (9)

where, $\hat{\Theta }$ represents the maximum likelihood estimation of the parameters **Θ** and *k* is freedom degrees of the parameter vector **Θ**. *N* is the number of samples and $f(Χ|\hat{\theta })$ indicates the joint probability density of *X*. The first term of the right end denotes model parameter estimation function. The second term represents the penalty function. When MDL are applied as source number estimates criteria, the model is expressed as follows[37]

$$MDL(k)=-N(M-k)\text{log}f(k)+\frac{1}{2}k(2M-k)\text{log}N$$ (10)

where

$$f(k)={\left(\prod_{i=k+1}^{M}\lambda_{i}\right)}^{\frac{1}{M-k}}(\frac{1}{M-k}\sum_{i=k+1}^{M}\lambda_{i})$$ (11)

And the number of the source can be yielded by minimizing the function of MDL.

$${\hat{q}}_{MDL}=\underset{k}{\text{arg min}}MDL(k)$$ (12)

The method of MDL estimates the source number by the difference of eigenvalues between the signal and the noise. Its computational complexity is relatively low, but it cannot process the signal with colored noise.

This paper brings in the diagonal loading technique to overcome the influence of the colored noise [38–40].

$$\beta_{i}=\lambda_{i}+\lambda_{DL},i=1,2,\ldots ,p$$ (13)

where, *λ*_*i*_, *i* = 1, 2, …, *p* are the original eigenvalues of *R*_*X*_, and *λ*_*DL*_ is the loading value. *β*_*i*_ is the final eigenvalues with diagonal loading. Diagonal loading process produces little effect to the bigger eigenvalues, which are corresponding to source signals. And the smaller eigenvalues corresponding to the noise will converge to near the loading value *λ*_*DL*_. Therefore, by diagonal loading process, the noise eigenvalues are approximately equal and the effect of this process is equivalent to whitening the colored noise.

The choice of loading value *λ*_*DL*_ has a great influence on this method. A small value of *λ*_*DL*_ plays little improvement to the estimation and an oversize *λ*_*DL*_ may destroy the difference of eigenvalues between noise and signal.

Therefore, the loading value *λ*_*DL*_ should satisfy the follow equations.

$$\lambda_{N}<<\lambda_{DL}<<\lambda_{S}$$ (14)

where *λ*_*N*_ relates to the noise eigenvalue, and *λ*_*S*_ represents to the signal eigenvalue. To meet this condition, a feasible selection of *λ*_*DL*_ is described as[38]

$$\lambda_{DL}=\sqrt{\sum_{i=1}^{p}\lambda_{i}}$$ (15)

Then the eigenvalues of *R*_*X*_ are transformed to follow by diagonal loading.

$$\beta_{i}=\lambda_{i}+\sqrt{\sum_{i=1}^{p}\lambda_{i}},i=1,2,\ldots ,p$$ (16)

### GDE method

For a *p* × *p* matrix *R*, *r*_*ij*_ is the element of row *i* and column *j*. The sum of the other elements in the *i* row except the *i* element is defined as

$$C_{i}=\sum_{j=1,j\neq i}^{L}\left|r_{ij}\right|i=1,\ldots ,p$$ (17)

The *i*th disk represents the circle with center of *r*_*ij*_ and radius of *C*_*i*_ in the complex plane.

$$O_{i}=\left\{z|\left|z-r_{ii}\right|\leq C_{i}\right\},i=1,\ldots ,p$$ (18)

Gerschgorin has proven that eigenvalues of matrix *R* are included in the region of disk *O*_*i*_, *i* = 1, …, *p*. So that the eigenvalues of *R* satisfying at least one of the following inequality.

$$\left|\lambda -r_{ii}\right|\leq C_{i},i=1,2,\ldots ,p$$ (19)

The signal covariance matrix is expressed as follow by EVD.

$$R=U\Lambda U^{H}$$ (20)

Where *U* is a *p*–*order* unitary matrix constructed by eigenvectors of the covariance matrix R, *U* = [*u*_1_, *u*_2_, …, *u*_*p*_], *UU*^*H*^ = *I*. **Λ** is a p-order diagonal matrix whose diagonal elements are the eigenvalues of matrix R, **Λ** = *diag* [**λ_1_**, …, **λ**_*p*_]. *R* can be rewrote in the form of block matrix.

$$R=\left(\begin{array}{cccc} r_{11} & r_{12} & \cdots & r_{1p}\\ r_{21} & r_{22} & \cdots & r_{2p}\\ \vdots & \vdots & \ddots & \vdots \\ r_{p1} & r_{p2} & \cdots & r_{pp} \end{array}\right)=\left(\begin{array}{cc} R_{1} & r\\ r^{H} & r_{pp} \end{array}\right)$$ (21)

Among them, *R*_1_ is a *p*– 1 order submatrix obtained by deleting the last row and column of R; *r* is a column vector constructing by the former *p*– 1 elements in the *p* column of R, that is *r* = [*r*_1*p*_,…, *r*_(*p*-1)*p*_]^*T*^. *R*_1_ can be transformed as follow by EVD.

$$R_{1}=U_{1}\Lambda_{1}U_{1}^{H}$$ (22)

where, *U*_1_ is a *p*–1 order unitary matrix composed by the eigenvectors of *R*_1_, $U_{1}=\left[u_{1}^{\prime },\ldots ,u_{p-1}^{\prime }\right]$. **Λ**_1_ is a diagonal matrix with eigenvalues of *R*_1_ being the diagonal elements, $\Lambda_{1}=diag\left[\lambda_{1}^{\prime },\ldots ,\lambda_{p}^{\prime }\right]$, where the eigenvalues are sorted in descending order. The relationship between the eigenvalues of *R*_1_ and *R* can be written as follow.

$$\lambda_{1}\geq \lambda_{1}^{'}\geq \lambda_{2}\geq \lambda_{2}^{'}\geq \cdots \geq \lambda_{p-1}\geq \lambda_{p-1}^{'}\geq \lambda_{p}$$ (23)

Then a *p* order matrix *U*_*d*_ is constructed.

$$S=U_{d}^{H}RU_{d}=\left(\begin{array}{cc} U_{1}^{H}RU_{1} & U_{1}^{H}r\\ r^{H}U_{1}^{} & r_{pp} \end{array}\right)=\left(\begin{array}{cc} \Lambda_{1} & U_{1}^{H}r\\ r^{H}U_{1}^{} & r_{pp} \end{array}\right)=\left(\begin{array}{cccc} \lambda_{1}^{\prime } & 0 & \cdots & 0\\ 0 & \lambda_{2}^{\prime } & \cdots & 0\\ \vdots & \vdots & \ddots & \vdots \\ \begin{array}{l} 0\\ \rho_{1}^{*} \end{array} & \begin{array}{l} 0\\ \rho_{2}^{*} \end{array} & \begin{array}{l} \cdots \\ \cdots \end{array} & \begin{array}{l} \lambda_{p-1}^{\prime }\\ \rho_{p-1}^{*} \end{array} \end{array}\begin{array}{c} \begin{array}{l} \rho_{1}\\ \rho_{2} \end{array}\\ \vdots \\ \rho_{p-1}\\ r_{pp} \end{array}\right)$$ (24)

$$U_{d}=\left(\begin{array}{cc} U_{1} & 0\\ 0^{T} & 1 \end{array}\right)$$ (25)

Transformed covariance matrix *R* with *U*_*d*_.

From the above equation, the radius of the former *p*– 1 Gerschgorin’s disk of matrix S can be written as

$$C_{i}=\left|\rho_{i}\right|=\left|u_{i}^{\prime }{}^{H}A_{1}R_{s}\eta_{p}^{*}\right|,i=1,\ldots ,p-1$$ (26)

The noise eigenvectors $u_{i}^{\prime },i=q+1,\ldots ,p-1$ of matrix *R*_1_ are orthogonal with the column vectors of *A*_1_, so *C*_*i*_ = 0, *i* = *q* +1, …, *p* − 1. But the signal eigenvectors are not orthogonal with *A*_1_ and the *R*_*s*_ is full rank matrix, so *C*_*i*_ ≠ 0, *i* = 1, …, *q*. Therefore, the covariance matrix *S* can be further simplified.

$$\text{S}=\left(\begin{array}{ccccccc} \lambda_{1}^{\prime } & 0 & & \cdots & & 0 & \rho_{1}\\ 0 & \ddots & & & & & \vdots \\ & & \lambda_{q}^{\prime } & & & \vdots & \rho_{q}\\ \vdots & & & \sigma^{2} & & & 0\\ & & & & \ddots & 0 & \vdots \\ 0 & & \cdots & & 0 & \sigma^{2} & 0\\ \rho_{1}^{*} & \cdots & \rho_{q}^{*} & 0 & \cdots & 0 & r_{pp} \end{array}\right)$$ (27)

The Gerschgorin disks of *S* are split into two groups. The one with nonzero radius and larger center point is belong to the actual signal and the others is corresponding to the noise. Therefore, the number of nonzero Gerschgorin disk radius in *S* is equal to the estimated source number. In practical applications, due to the limited number of snapshots, there will be some bias and the disk radiuses of noise are not always zero. So GDE method is put in place to determine the number of sources.

$$GDE(k)=C_{k}-\frac{D(N)}{p-1}\sum_{i=1}^{p-1}C_{i},k=1,\ldots ,p-1$$ (28)

where, *D*(*N*) is the adjustment factor with a value between 0 and 1. Calculating Eq (27) from *k* = 1 to *k* = *p*, when *GDE*(*k*) come up with negative value first time, the calculation will be stopped and the estimated number of sources is *k* − 1.

The GDE method gets the source number by utilizing the size of the radius of the transformed covariance matrix. Comparing with methods that based on ITC, it has certain superiority of keeping a well-behaved in the condition of colored noise.

However, the GDE method is restricted by its poor performance at low SNR. This paper introduced jackknife technique to optimize the data covariance matrix. Then the influence of noise can be reduced to enhance the performance of GDE at low SNR.

Jackknife technique is an effective strategy utilized in the statistical area. It can take full advantage of the received data to achieve more accurate detection and estimation [20]. For jackknife, the data structure is reconstructed by deleting a part of data every time and it can reduce the bias of the estimator.

A N × *d* data matrix has been obtained from a 1 × *L* single-channel data by delay process.

$$Y=\left[\begin{array}{c} y(1),y(2),\ldots ,y(d)\\ y(d*1+1),y(d*1+2),\ldots ,y(d*1+d)\\ \vdots \\ y(d*(N-1)+1),y(d*(N-1)+2),\ldots ,y(d*(N-1)+d) \end{array}\right]=\left[\begin{array}{c} Y_{1}\\ Y_{2}\\ \vdots \\ Y_{N} \end{array}\right]$$ (29)

where, *Y* is regarded as a set with *N* elements, *Y* = [*Y*_1_, *Y*_2_, …, *Y*_*N*_]^*T*^. *M* elements are chosen randomly from *Y* to form a subset *X*.

$$X={\left[X_{1};X_{2};\ldots ;X_{M}\right]}^{T}$$ (30)

Where *X* ⊂ *Y*, *X*_*j*_ ∈ *Y*, *j* = 1, 2, …, *M*, and *M* = *rN*, 0.5 ≤ *r* < 1. $\bar{R}$ is the covariance matrix of *X*.

$$\bar{R}=X\cdot X^{H}$$ (31)

Repeating the above process *K* times, a series of covariance matrixes are obtained, *R*_*j*_, *j* = 1, 2, …, *K*. Calculate the average of these matrices.

$${\bar{R}}_{a}=\sum_{j=1}^{K}R_{j}/K$$ (32)

where, ${\bar{R}}_{a}$ is the optimized covariance matrix, which will be used to estimate the source number by GDE.

## Experimental Studies

This section gives some experimental results to evaluate the performance of each source number estimation method mentioned above.

**Fig 1:**
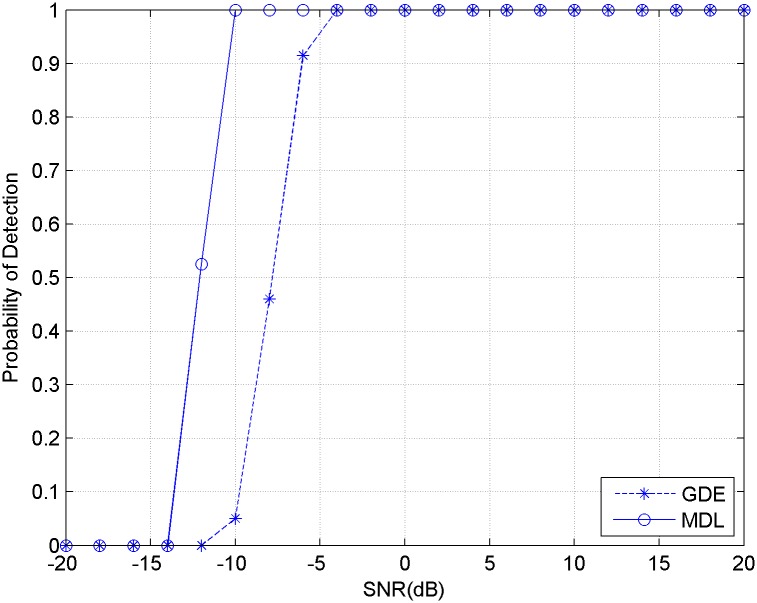
Detection probability of MDL and GDE for signals with white noise.

The first experiment compares the estimation performance of MDL and GDE when the received signal is contained with white Gaussian noise. Assuming that the single channel received signal appears with 3 independent source signals, which are constructed in Matlab environment. The observation data length is set to be *L* = 5000, and the delay is *d* = 600. Through the use of delay process, the number of virtual channel is *M* = 8. Consequently, the single-channel data is expanded to a 8×600 data matrix. The SNR of the received signal is changed from -20dB to 20dB, with 2dB increments. The experiment with each condition is repeated with 500 times. The first experiment result is shown in Fig 1.

It can be inferred from Fig 1 that both MDL and GDE methods achieve a good estimation performance with white Gaussian noise. However, due to the fact that the Gerschgorin disk radius are influenced by noise relatively easy, the performance of GDE method is slightly worse than that of MDL at low SNR.

**Fig 2:**
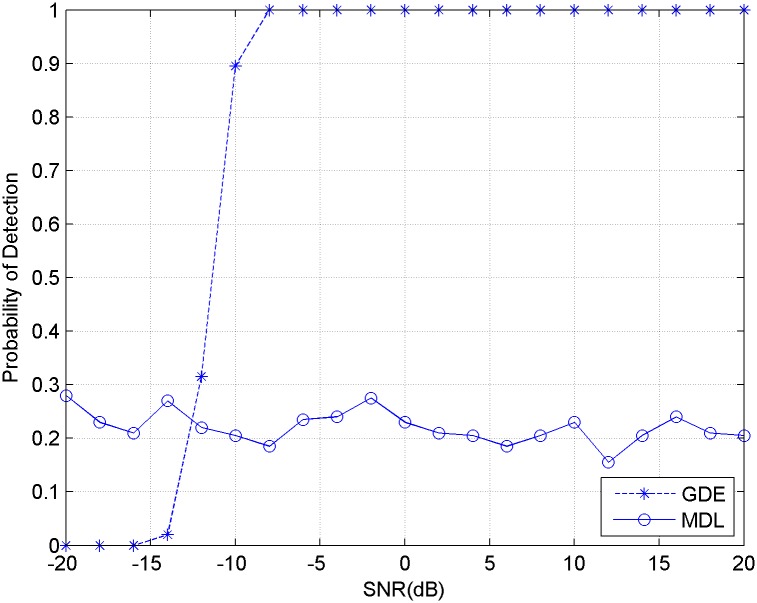
Detection probability of MDL and GDE for signals with colored noise.

The second experiment compared the estimation performance of MDL and GDE with colored noise. The condition of this experiment is the same as the first one, except that the white noise is replaced with colored noise. The 500 experimental results of Monte Carlo are provided in Fig 2. It shows that the method of MDL has lost its estimation ability with colored noise, but GDE method still keeps a good performance.

**Fig 3:**
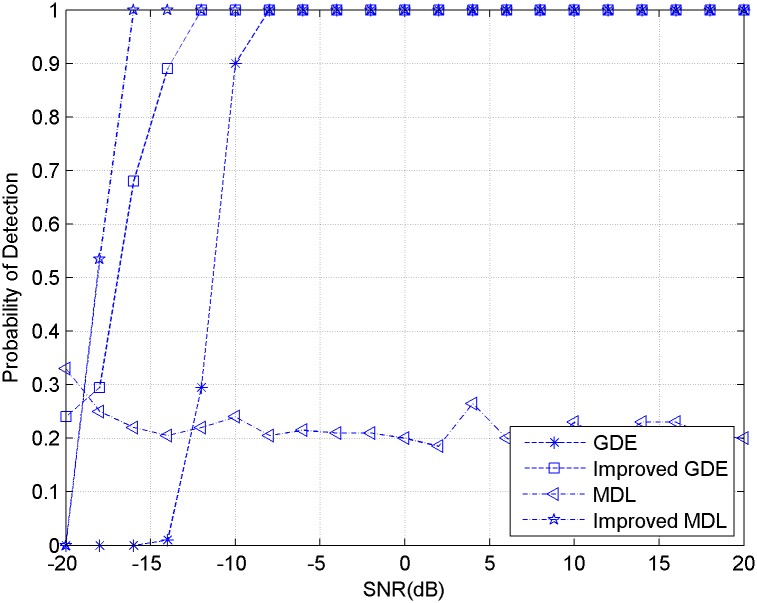
Detection probability of original and improved methods for signals with colored noise.

The third experiment compared the estimation performance of MDL, improved MDL, GDE and improved GDE with colored noise. The experimental condition is set the same as the first and second ones. Fig 3 shows the detection probability of these methods under different SNR.

It can come to conclusion that the boosting put forward by this paper makes the performance of MDL and GDE methods get improvement largely. The improved MDL method proposed by this paper can eliminate the effect of colored noise. The GDE method keeps a superior performance at low SNR.

## Conclusion

It is a widespread and challengeable problem to enumerate the source number of single channel received data in many fields. For example, source number means the number of defects in material under testing in nondestructive testing such as the multi fatigue cracks in rail [41]. Therefore it should be estimated accurately for further defect quantification [42]. This paper investigates the MDL and GDE methods to solve this problem. In order to effectively utilize the algorithms used in array signal processing, the single channel received data is transformed into multi-dimension form by delay process and the data covariance matrix is constructed. The main contribution of this paper is that diagonal loading technique referenced to optimize the MDL method. Therefore, it can eliminate the influence of colored noise. Another achievement of this paper is employing the jackknife technique to optimize the performance of the GDE method at low SNR. The experimental results proved that the improved MDL method can eliminate the effect of colored noise. Furthermore, the improved GDE method obtains a superior performance than the original one.
